# 

**Published:** 1902-08

**Authors:** 


### CONTENTS.

ORIGINAL ARTICLES:

Investigation of the Pathogenesis of Roaring (Hemiplegia Laryngis). By PROFESSOR M. H. J. P. THOMASSEN. Translated by L. VAN ES, M.D., V.S. . . . . . . . 469

The Value of Co-operation in the Sanitary Control of Our Periodic Epizoötics of Anthrax. By W. H. DALRYMPLE, M.R.C.V.S. . . . . . . . . . . . . . . 484

Notes on a Feeding Experiment to Produce Leucoencephalitis in a Horse, with Positive Results. By TAIT BUTLER, V.S. . . . . . . . . . . . . . 498

Ectropion and Entropion. By S. H. Swain, V.S. . . . . . . . . . . 501

ABSTRACTS FROM FOREIGN JOURNALS . . . . . . . . . . . . . . 504

REPORTS OF CASES:

A Case of Hæmatic Filariasis in a Dog By CHARLES F. DAWSON, V.S. . . . 507

EDITORIALS:

The Penalty of Neglecting Veterinary Medicine . . . . . . . . . . 508

Another Danger Signal Out . . . . . . . . . . . . . . 509

Another School in the Field . . . . . . . . . . . . . . 510

Alternating Currents . . . . . . . . . . . . . . 510

NECROLOGY . . . . . . . . . . . . . . . . . . . 510

SOCIETY PROCEEDINGS—COMMENCEMENT EXERCISES—AMONG THE COLLEGES—BOOK NEWS—NOTES—PERSONALS . . . . . . . . . . . . . 512–536

